# Norwegian society of rheumatology recommendations on diagnosis and treatment of patients with giant cell arteritis

**DOI:** 10.3389/fmed.2022.1082604

**Published:** 2023-01-06

**Authors:** Anne Bull Haaversen, Lene Kristin Brekke, Gunnstein Bakland, Erik Rødevand, Geirmund Myklebust, Andreas P. Diamantopoulos

**Affiliations:** ^1^Department of Rheumatology, Martina Hansens Hospital, Bærum, Norway; ^2^Department of Rheumatology, Hospital for Rheumatic Diseases, Haugesund, Norway; ^3^Department of Rheumatology, University Hospital of Northern Norway, Tromsø, Norway; ^4^Department of Rheumatology, St. Olav’s University Hospital, Trondheim, Norway; ^5^Department of Research, Hospital of Southern Norway, Kristiansand, Norway; ^6^Department of Infectious Diseases, Division of Internal Medicine, Akershus University Hospital, Lørenskog, Norway

**Keywords:** giant cell arteritis (GCA), large vessel vasculitis, guidelines, diagnosis, treatment

## Abstract

**Objective:**

To provide clinical guidance to Norwegian Rheumatologists and other clinicians involved in diagnosing and treating patients with giant cell arteritis (GCA).

**Methods:**

The available evidence in the field was reviewed, and the GCA working group wrote draft guidelines. These guidelines were discussed and revised according to standard procedures within the Norwegian Society of Rheumatology. The European Alliance of Associations for Rheumatology (EULAR) recommendations for imaging and treatment in large vessel vasculitis and the British Society for Rheumatology (BSR) guidelines for diagnostics and treatment in GCA informed the development of the current guidelines.

**Results:**

A total of 13 recommendations were developed. Ultrasound is recommended as the primary diagnostic test. In patients with suspected GCA, treatment with high doses of Prednisolone (40–60 mg) should be initiated immediately. For patients with refractory disease or relapse, Methotrexate (MTX) should be used as the first-line adjunctive therapy, followed by tocilizumab (TCZ).

**Conclusion:**

Norwegian recommendations for diagnostics and treatment to improve management and outcome in patients with GCA were developed.

## Introduction

### Background

Giant cell arteritis (GCA) is the most common systemic vasculitis in adults and has a spectrum of possible presentations (1, 2). The main subsets include isolated cranial arteritis (c-GCA), isolated large vessel vasculitis (LV-GCA), and coexisting cranial and large vessel vasculitis (mixed-GCA) (2). Polymyalgia rheumatica (PMR) and GCA are closely related, and some argue that PMR lies within the spectrum of GCA. However, these conditions may occur simultaneously or independently of each other (3–5).

Giant cell arteritis occurs almost exclusively in people older than 50 years of age, with a peak in onset between 70 and 80 years of age and with a female predominance (6, 7). The majority of epidemiological studies on GCA have investigated European or North American populations, and the highest incidence has been reported in Nordic countries or North-American people of Scandinavian descent (8). Data on GCA occurrence in Africa, Asia, the Middle East, South- and Latin America, and Oceania are sparse and suggest that the condition is less common in non-Caucasians.

The annual incidence rate for GCA in Norway was recently estimated to be 22.5 per 100,000 persons ≥50 years of age (7).

Giant cell arteritis’ etiopathogenesis is not fully understood but is considered to involve a combination of genetic and environmental factors (9). Different compositions of genetic background may contribute to global differences. There is also evidence that lifestyle factors such as body mass index, glucose levels, and smoking may influence the risk of GCA (10–14).

### Objectives of the guidelines

These recommendations aim to provide clinical guidance to Rheumatologists and other clinicians involved in diagnosing and treating GCA patients. The guidelines cover individuals older than 50 years of age suspected to have GCA. The guidelines aim to harmonize the diagnostic and treatment procedures across specialists and departments in the Norwegian Healthcare System.

## Methods

The Norwegian GCA working group (the members are the authors of these guidelines) developed the recommendations by reviewing the available evidence and writing the draft guidelines. The draft guidelines were discussed and revised according to standard procedures within the Norwegian Society of Rheumatology.

The European Alliance of Associations for Rheumatology (EULAR) recommendations for imaging and treatment in large vessel vasculitis (15, 16) and The British Society for Rheumatology (BSR) guidelines for diagnostics and treatment in GCA (17) served as the basis to the development of current guidelines. As the American College of Rheumatology guidelines recommend the use of Tocilizumab very early in the treatment of GCA patients, which is not refunded by the Norwegian Health Authorities, we chose not to incorporate these guidelines in the Norwegian guidelines (18). Also, the evidence on diagnostics and treatment of GCA published after 2018 was reviewed and included in this work. The PubMed and a combination of the search terms Giant cell arteritis and treatment and/or diagnosis were used. The review of individual studies was restricted to randomized controlled studies or prospective observational studies with >50 participants. The guidelines were proposed, discussed, revised, and accepted by voting/reaching an agreement by the majority of the members of the working group and the professional council (in Norwegian: Fagrådet) of the Norwegian Society of Rheumatology. The method used to obtain consensus was voting among members during meetings of the working group.

The present guidelines are the foundation upon which clinical practice should be based. Guidelines, unlike some types of policies, are not mandatory. Individual patient circumstances may influence clinical decisions, and clinicians should work alongside patients to make care-based shared decisions. Thus, failure to adhere to these guidelines should not necessarily be considered negligent. These guidelines should not be used to limit access to other diagnostic or treatment options.

## Results

### Diagnosis

#### Recommendation 1

In patients with suspected GCA, a thorough history should be obtained, including inquiry about polymyalgia symptoms, new-onset headache, tongue- or jaw-claudication, vision disturbances, arm or leg claudication, constitutional symptoms (fatigue, fever, and weight loss). Additionally, a thorough clinical examination and laboratory workup should be performed including heart and lung auscultation, blood pressure in both arms, temperature, C-reactive protein (CRP), erythrocyte sedimentation rate (ESR), and full blood count (Table 1).

**TABLE 1:** Clinical symptoms and findings and laboratory results in giant cell arteritis (GCA) patients.

Clinical symptoms	Clinical findings	Laboratory findings
New-onset headache, often in the temporal area. Jaw/tongue claudication. Acute visual symptoms (e.g., amaurosis fugax, acute visual loss, diplopia) Constitutional symptoms (e.g., weight loss >2 kg, fever, fatigue, night sweats, and dry cough). Polymyalgia symptoms Limb claudication	Tenderness/thickening of the temporal arteries with or without reduced pulsation. Scalp tenderness Bruits (particularly in the axilla). Reduced pulses/blood pressure of the upper limbs	Anemia Elevated CRP or ESR Thrombocythemia Elevated liver enzymes (ALP) Normal creatinine

#### Recommendation 2

Patients suspected of having GCA, should be directly referred to a Fast-Track Clinic (FTC) or a rheumatologist for further evaluation, treatment, and follow-up within 24 h. In cases where FTC- or rheumatology consult is unavailable within 24 h the patient may be referred to other relevant specialists (e.g., neurologist, ophthalmologist, and internist). FTC consists of an evaluation by ultrasound or other imaging modality which can confirm the diagnosis immediately (19). Diagnostic work-up should not delay the initiation of treatment.

#### Recommendation 3

In patients with suspected GCA, ultrasound of at least temporal and axillary arteries (20) should be performed by an ultrasonographer experienced in vascular ultrasound using high-end ultrasound equipment (21) (Tables 2, 3). Ultrasound of the facial artery further increases the sensitivity to diagnose GCA (20). If ultrasound is not available or inconclusive, a biopsy of the temporal artery should be considered. The length of the biopsy should be >1 cm after fixation. Giant cells in the biopsy are not obligate for the diagnosis of GCA, but appropriate pathological findings should be present (Table 4). Alternatively, Magnetic Resonance Angiography (MRA) of temporal arteries or Positron Emission Tomography (PET-CT) may be performed. In addition, ultrasound of large vessels (carotid, vertebral, and subclavian) (22) or CT of large vessels or MRA or PET-CT depending on the availability is recommended. It is essential to recognize the disease extent as early as possible as large vessel involvement may indicate difficult-to-treat disease, while temporal artery involvement has been associated with a higher risk for visual loss (23, 24). The choice of imaging modality depends on the local availability.

**TABLE 2:** Ultrasonographic findings in giant cell arteritis (GCA) patients (33).

Vessels	Ultrasound
Cranial arteries	Halo sign: homogenous, hypoechoic wall thickening that is well delineated toward the luminal side that is visible both in longitudinal and transverse planes, most commonly concentric Compression sign: the thickened arterial wall remains visible upon compression due to vasculitic wall thickening in comparison to surrounding tissue
Large vessels Other findings	Most commonly concentric vessel wall thickening homogeneous, hypo- or isoechoic (increased Intima media thickness)’ Atherosclerosis hypo-, iso- or hyperechoic, non-homogeneous and localized plaques seen mainly in the large vessels at bifurcations

**TABLE 3:** Threshold intima media thickness (IMT) values in ultrasound examination (34).

Examined artery	IMT threshold (in mm)
Common temporal	0.42
Frontal temporal	0.34
Parietal temporal	0.29
Facial artery	0.37
Axillary artery	1.0
Subclavian artery	1.0
Occipital artery	0.4
Vertebral artery	0.7
Common carotid	1.0

**TABLE 4:** Histological findings in patients with giant cell arteritis (GCA) (35).

Histological features In GCA	
Location	All three arterial layers may be involved. In severe cases there is a diffuse widespread inflammatory infiltration Main inflammatory bulk is located in the adventitia media junction The inflammatory infiltrate has a concentric ring appearance, with the thicker ring adjacent to adventitia-media junction and the thinner ring in proximity to media-intima junction (transmural inflammation) The media is relatively spared The myofibroblastic proliferation of intima leads to occlusion of the lumen
Types of cells	CD-4+ lymphocytes and macrophages are the most commonly seen Giant cells are seen in 50–75% of cases and their absence do not preclude the diagnosis Plasma cells and eosinophils may also be seen; neutrophils are rarely present
**Other histological patterns**	
Periadventitial and adventitial inflammation Isolated intima inflammation	The inflammation in GCA spreads from adventitia to intima. Inflammation affecting only the periadventitial vessels, the vasa vasorum and the adventitial tissue may also be seen Cautious interpretation is needed taking into account clinical, laboratorial and imaging findings Rarely seen
Healed arteritis Atherosclerosis Fibrinoid necrosis	Features of healed inflammation: irregular intima proliferation, changes in the internal elastic lamina, fibrosis and neovascularization of the media and adventitia in the absence of ongoing active inflammation Note that: These changes can also be seen as a result of normal aging (atherosclerosis) Regular intima proliferation with focal loss of the internal elastic lamina. Calcifications could be present Rarely seen (Evaluate for other diagnoses, if clinical findings are not typical for GCA)

#### Recommendation 4

In a patient with high clinical suspicion of GCA and a positive diagnostic test (biopsy or imaging modality), no further test is required to confirm the diagnosis. In patients with low clinical suspicion of GCA and a negative diagnostic test (biopsy or imaging modality), the probability for GCA is low. In other cases, an individual assessment will be necessary for further diagnostics.

#### Recommendation 5

**FIGURE 1:**
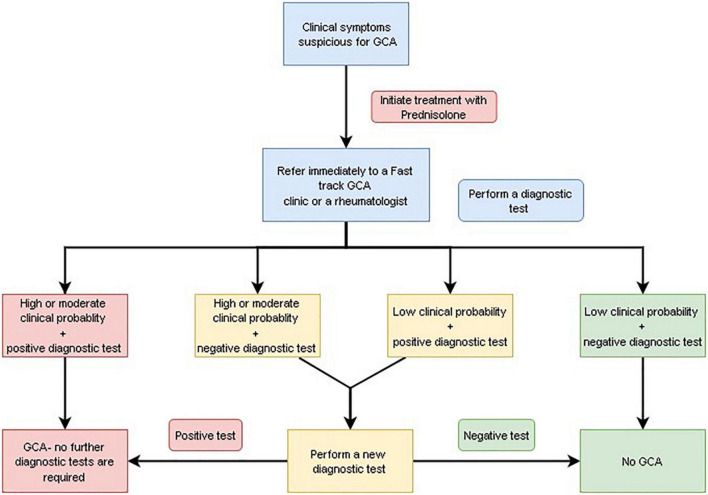
Diagnostic algorithm for giant cell arteritis (GCA).

In patients with GCA and visual symptoms, referral to an ophthalmologist is highly recommended. Initiation of treatment should not be delayed while waiting for this evaluation (Figure 1 and Table 5).

**TABLE 5:** Summary of the Norwegian society of rheumatology’s recommendations on diagnosis and treatment of patients with giant cell arteritis (GCA).

# of recommendation	
1	Refer patients suspected of having GCA to a Fast-Track GCA clinic (19) or a rheumatologist within 24 h. Treatment should not be delayed while waiting for this evaluation.
2	Obtain a thorough history and perform clinical examination and laboratory work up.
3	In patients with high clinical suspicion of GCA and a positive diagnostic test (temporal artery biopsy or any imaging modality) no further test is required to confirm the diagnosis.
4	Perform ultrasound of temporal and axillary arteries using high-end ultrasound equipment. Ultrasound of facial artery increases the sensitivity (32). If ultrasound is not available or inconclusive, perform another diagnostic test.
5	Refer to ophthalmologist if visual manifestations.
6	Initiate treatment with 40 mg Prednisolone/day in patients without visual manifestations. Initiate treatment with Prednisolone 60 mg/day if visual manifestations are present, consider a single dose of 500 mg IV methylprednisolone.
7	Taper daily Prednisolone dose as described in Table 6.
8	In minor relapse: Increase Prednisolone dose to the most recent effective dosage. In refractory disease or major relapse: Initiate Methotrexate (MTX) 20 mg/week sc. Consider Tocilizumab (TCZ) 162 mg/week sc if the patient is not tolerating or has a refractory or relapsing disease while on MTX.
9	Patients with GCA and high risk for osteoporosis should receive treatment according to the Norwegian guidelines for osteoporosis diagnostics and treatment.
10	Acetylsalicylic acid should not be used routinely, and should be considered on individual indication.
11	A relapse should be confirmed by an imaging modality. Modified Kerr’s (NIH criteria) could be used to monitor disease activity (31).
12	Reevaluate the diagnosis in patients not responding to standard treatment.
13	Follow-up should be performed every month until remission is achieved, and then after 3 months, 6 months, and yearly.

### Treatment

#### Recommendation 6

In patients with suspected GCA, glucocorticoids should be immediately initiated. The diagnostic work-up should not delay the initiation of treatment. For patients without visual manifestations we recommend a starting dose of 40 mg Prednisolone/day. In patients with visual involvement (visual loss, diplopia, amaurosis fugax, and blurred vision), we recommend starting with 60 mg Prednisolone/day. A single dose of 500 mg × 1 methylprednisolone iv followed by Prednisolone 60 mg/day may be individually considered, but the evidence is sparse (25–27).

#### Recommendation 7

In patients with GCA, Prednisolone should be tapered by 5 mg every 2nd week till 20 mg/day, after that with 2.5 mg every 3rd week till 10 mg/day, and after that with 1.25 mg every 3rd week to 5 mg/day. We recommend continuing treatment with 5 mg/day at least for 1 year after initiation of Prednisolone. Further tapering can be considered on an individual basis if the patient has been in remission for at least 1 year. When starting with dose 60 mg/day, Prednisolone should be tapered by 10 mg every week till 40 mg/day, thereafter tapering as described above (Table 6).

**TABLE 6:** Prednisolone tapering.

Weeks	Prednisolone dose (mg)	
1	60	Starting dose when visual manifestations
2	50	
3 + 4	40	Starting dose when no visual manifestations
5 + 6	35	
7 + 8	30	
9 + 10	25	
11 + 12	20	
13 + 14 + 15	17.5	
16 + 17 + 18	15	
19 + 20 + 21	12.5	
22 + 23 + 24	10	
25 + 26 + 27	8.75	
28 + 29 + 30	7.5	
31 + 32 + 33	6.25	
34–52	5	

#### Recommendation 8

If the patients suffers a relapse, we recommend an increase in Prednisolone dose to the last effective dose, or a higher dose based on the severity of the relapse and an individual assessment. In patients with refractory disease or a major relapse (Table 7), initiation of Methotrexate (MTX), preferably subcutaneously, 20 mg/week, should be considered. The dose should be adjusted according to the patient’s age and kidney function. Tocilizumab (TCZ) 162 mg/week sc should be considered if the patient is not tolerating MTX or suffer a relapse while on MTX (according to the Norwegian Tender System). Leflunomide or Azathioprine may be considered, but evidence supporting their use is scarce. There is currently no robust evidence supporting the use of TNF-α inhibitors or other biologics than TCZ in patients with GCA.

**TABLE 7:** Definitions of disease activity (16).

Major relapse	Recurrence of active disease with either of the following: a. Clinical features of ischemia (jaw claudication, visual symptoms, visual loss attributable to GCA, scalp necrosis, stroke, limb claudication) b. Evidence of active aortic inflammation resulting in progressive aortic or large vessel dilatation, stenosis, or dissection
Minor relapse	All patients suffering a relapse without the characteristics of a major relapse (constitutional symptoms, polymyalgia, and headache)
Refractory disease	Active disease despite the use of standard care therapy
Remission	Absence of all clinical signs and symptoms attributable to active GCA Normalization of ESR and CRP No evidence of progressive vessel narrowing or dilatation for patients with large vessel involvement

#### Recommendation 9

Giant cell arteritis patients with new-onset disease should be referred to a measurement of bone mass density. In patients with GCA and high risk for osteoporosis, treatment according to the Norwegian guidelines for osteoporosis should be initiated (28).

#### Recommendation 10

**FIGURE 2:**
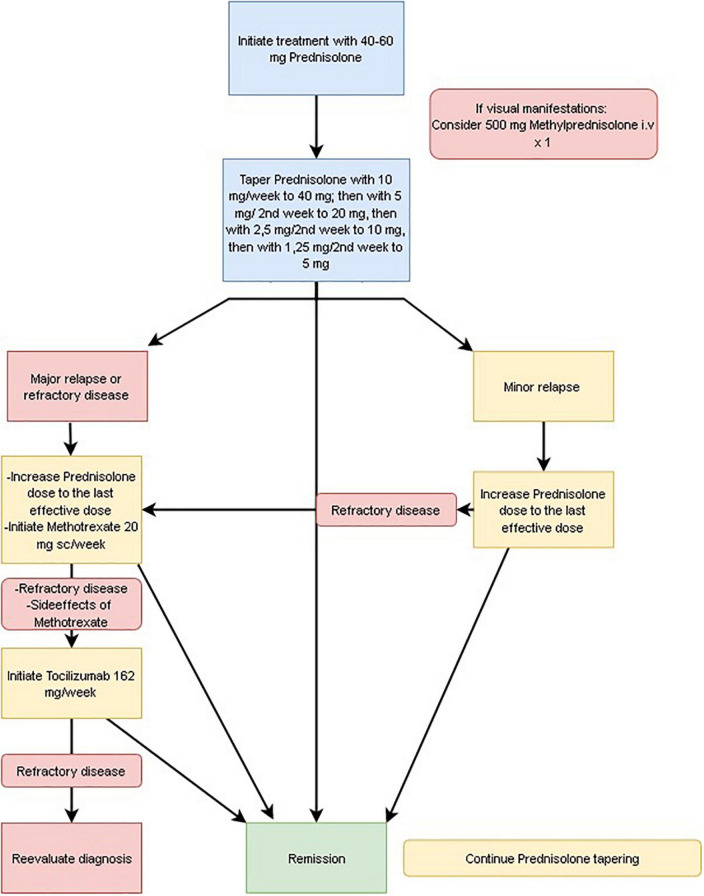
Treatment algorithm for giant cell arteritis (GCA).

In patients with GCA, we do not routinely recommend using Acetylsalicylic acid unless cardiovascular reasons support its use (Figure 2).

### Follow up

#### Recommendation 11

In GCA patients, follow-up should be continued every month until remission is achieved. Thereafter follow-up at 3 months, 6 months, and thereafter yearly.

#### Recommendation 12

In GCA patients who suffer a relapse, the relapse should, if possible, be confirmed by an imaging modality, preferably ultrasound, and/or laboratory tests (29, 30). Modified Kerr’s (NIH) criteria (originally developed for Takayasu arteritis) may be used to monitor disease activity (31) (Tables 7, 8).

**TABLE 8:** Modified Kerr’s criteria: >1 point indicates active disease (31).

Elevated CRP or ESR not attributed to other causes than vasculitis Clinical symptoms of ischemia (headache and jaw claudication) not attributed to other causes than vasculitis	+1 +1
Constitutional symptoms (fatigue, fever, weight loss, and polymyalgia symptoms) not attributed to other causes than vasculitis Findings suggesting active vasculitis in an imaging modality: Involvement of new vascular areas Increasing IMT in already involved areas	+1 +1

#### Recommendation 13

In GCA patients with relapsing or refractory disease, alternative diagnoses should be considered (e.g., malignancy, autoinflammatory syndromes).

## Data availability statement

The original contributions presented in this study are included in the article/supplementary material, further inquiries can be directed to the corresponding author.

## Author contributions

AH prepared the manuscript. AD, LB, GB, GM, and ER contributed equally to the manuscript. All authors contributed to the article and approved the submitted version.
